# Novel agents and evolving strategies in myelofibrotive neoplasm: an update from 2022 ASH annual conference

**DOI:** 10.1186/s13045-023-01446-0

**Published:** 2023-05-12

**Authors:** Andrew Wang, James Liu, Jeffrey J. Pu

**Affiliations:** 1 Pennsylvania State University, University Park, PA 16802 USA; 2 Hopkins School, 986 Forest Road, New Haven, CT 06515 USA; 3 Boston VA Medical Center/Brigham and Woman Hospital, Harvard Medical School, 1400 VFW Parkway Building 3, Suite 2B-115, Boston, MA 02132 USA

**Keywords:** Myelofibrotive neoplasm, Targeted therapy, Clinical research

## Abstract

Myelofibrosis (MF) is a disorder characterized by the proliferation of myeloid precursors, commonly due to overactive JAK signaling. The discovery of the JAK2^V617F^ mutation and subsequent development of JAK inhibitors (JAKi) results in reduced spleen size, improved symptom, and enhanced survival in MF patients. However, there are unmet needs of additional novel targeted therapies for this incurable disease due to the limited utility of first-generation JAKis, which are associated with dose-limiting cytopenia and disease recurrence. New targeted treatment strategies for MF are on the horizon. We are here to discuss the latest clinical research findings presented in the 2022 ASH Annual Meeting.

## To the editor

Myelofibrosis research has dramatically advanced in the last several years [1]. In this article, we summarized some of the most exciting developments and innovations in investigational targeted therapeutic agents and novel regimens in treating MF from the 2022 ASH Annual Meeting.

## Targeted therapeutics of myelofibrosis

JAK2^V617F^ is the most common mutation, leading to the overactivation of JAK/STAT signaling linked with clonal expansion in myeloproliferative neoplasms (MPNs) [2]. JAK/STAT pathway inhibition has become the cornerstone therapeutic strategy for patients with symptomatic MF. Ruxolitinib (RUX), Fedratinib and Pacritinib are FDA approved JAK1/2 inhibitors for treatment of intermediate and high-risk MF [2]. Even though these drugs change the landscape of MF management and provide significant clinical benefits, approximately 1/3 of MF patients either cannot tolerate the treatment, developing cytopenias during treatment, or do not respond to the therapy well [2]. In abstract 627 and 3028, the phase III MOMENTUM data demonstrate that Momelotinib (MMB), the first JAK1/2/ACVR1 inhibitor, reduced symptoms and spleen volumes, improved transfusion independence (TI), and prolonged survival in a group of symptomatic and anemic MF patients who failed JAKi treatment (Table 1) [3]. Additional analysis also shows that transfusion independence response (TI-R) at W24 is a potential surrogate for improved overall survival (OS) (Table 1). In abstract 628, MF patients treated with Pacritinib, another FDA approved JAK2 inhibitor, achieved greater TI in comparing the ones with best-available therapy (BAT) (24% vs. 5%, based on SIMPLIFY criteria; 37% vs. 7%, based on Gale criteria) in a phase III PERSIST-2 trial, which enrolled MF patients with severe thrombocytopenia. The possible mechanisms of erythropoietic benefit of Pacritinib were inhibition of activin A receptor, type I (ACVR1) (Table 1) [4]. In abstract 240, TP-3654, a selective oral PIM1 Kinase Inhibitor, was utilized in an ongoing Phase I/II study showing encouraging signs of clinical activity in spleen volume reduction (SVR), symptom improvement, and cytokine reduction in MF patients previously treated with JAKi. TP-3654 is well tolerated with limited myelosuppressive adverse events (Table 1) [5]. ASXL1 mutations confer poor prognosis in MF patients with low JAK2^V617F^ allele burden. Abstract 4368 reports a Lysine-specific demethylase-1 (LSD1) inhibitor, IMG-7289, as an oral monotherapy, which reduced symptoms, SVR, BMF, TSS50 and selectively inhibited ASXL1 mutation clones with acceptable tolerability in a phase II study (Table 1) [6].

**Table 1:** Selected studies on the novel single agent targeted therapeutic on myelofibrosis from the 2022 ASH annual meeting

Name	Target	Route	Trial phase	Inclusion criteria (prior treatments; subject age; spleen size)	Subjects Baseline Characteristics	Study duration (current /planned)	Accrual (current accrual #/target accrual #)	Efficacy	Adverse Events	Clinicaltrials.gov Registration	References
MMB	JAK1/2/ACVR1	PO	III	One prior JAKi for ≥ 90 days; ≥ 18 years; palpable spleen ≥ 5 cm	N/A	27/96 months	195/195	TI-R (31%); SVR (100%) at W24	Thrombocytopenia Anemia Infection Peripheral neuropathy	NCT04173494	[3]
Pacritinib	JAK2/ACVR1	PO	III	JAKi-naïve patients; ≥ 18 years; palpable spleen ≥ 5 cm	All patients had platelet counts ≤ 100 × 10^9^/L	40/40 months	327/327	TL-R (24% on SIMPLIFY criteria, 37% on Gale criteria) at W24	Not reported	NCT01773187	[4]
TP-3654	PIM1	PO	I/II	At least one prior JAKi; ≥ 18 years; palpable spleen ≥ 5 cm or SV ≥ 450 cm^3^	Median age of 70 years; median spleen volume of 2370 cm^3^	32/55 months	8/60	SVR (83%); TSS50 (66%); at W12	Nausea Vomiting Diarrhea	NCT04176198	[5]
IMG-7289	LSD1	PO	II	Prior JAKi or JAKi-naïve patients; ≥ 18 years;	Median age of 68 years; median spleen volume of 1354 cm^3^	56/56 months	89/89	SVR (66%); TSS50 (19%); TI-R (90%); BMF (53%) at W24	Dysgeusia Diarrhea	NCT03136185	[6]

## Frontline “Add-on” targeted therapeutics of myelofibrosis

In the front-line setting, some novel targeted therapeutics are used in combination with RUX to improve the depth of response seen upfront with single agent RUX. In a pre-clinical study**,** Lu et al. shows that Siremadlin (SIR), an HDM2 inhibitor, was able to restore p53-mediated apoptosis in MF via combining with other pharmacological agents that disrupted the interplay between HDM2/p53, HIF1α and nuclear factor kappa B (NFκB) pathways [7]. Furthermore, abstract 239 reports an phase I/II ADORE study Part 1 (phase Ib), where the recommended phase 2 dose (RP2D) of SIR was established as 30 mg orally once daily on days 1–5/28-day cycle when added to the existing stable dose of RUX in patients who presented either persistent splenomegaly (spleen size ≥ 5 cm from the left costal margin or spleen volume ≥ 450 cm^2^ by MRI/CT scan) or continuous anemia (HgB < 11 g/dL) after at least 12 weeks of RUX monotherapy [8]. Good tolerability at 30 mg daily allowed patients to remain on SIR + RUX and to achieve robust SVR at W24 (Table 2). Pelabresib (PELA) is a selective Bromodomain and Extraterminal (BET) inhibitor to modify NFκB signaling related genes’ expression. In the MANIFEST phase II study, PELA combining with RUX (Arm 3) showed improved spleen volume reduction of 35% (SVR35) and total symptom score reduction of ≥ 50% (TSS50) and BMF improvement at any time (Table 2) in JAKi-naïve MF patients with intermediate-1/2 or high risks [9]. Navitoclax inhibits the anti-apoptotic BCL-2 family proteins (primarily BCL-XL). In the REFINE phase II study (Cohort 3; Abstract 237), navitoclax combining with RUX achieved SVR35 at W24 in all subgroups known to confer poor prognosis [10]. The paralleled reduction of both driver mutation JAK^V617F^’s VAF (36% patients achieved > 50% VAF reduction from baseline) and BMF indicates that this combination therapy regimen is promising (Table 2). In abstract 236, Parsaclisib, a potent and highly selective inhibitor of PI3 kinase, was assessed as an “add-on” agent to RUX among MF patients with suboptimal response to RUX in a phase II trial. The trial data show improvement in both symptoms and spleen size [11]. Responder efficacy variables analysis (SV, MF-SAF, and MPN-SAF-TSS) indicates that the continuous daily dosing regimen was more efficacious than daily dosing for 8 weeks then following with weekly dosing. This combination therapy was associated with limited grade 3/4 AEs and TEAE-related discontinuations (Table 2). Selinexor (SEL) is a Selective Inhibitor of Nuclear Export (SINE) compound that inhibits XPO1, which leads to nuclear retention and activation of tumor suppressor proteins. Abstract 1734 presents a phase I/II study evaluating the impact of SEL + RUX combination in treating JAKi-naïve MF. The preliminary data from this study demonstrate a manageable safety profile and encouraging preliminary data on SV, symptoms and TI-R (Table 2) [12].

**Table 2:** Selected studies on the novel targeted therapeutic in combination with RUX on myelofibrosis from the 2022 ASH annual meeting

Name	Target	Route	Trial Phase	Inclusion Criteria (prior treatments; subject age; spleen size)	Subject Baseline Characteristics	Study Duration (current /planned)	Accrual (current accrual #/target accrual #)	Efficacy	Adverse Events	Clinicaltrials.gov Registration	References
SIR	HDM2	PO	Ib	One prior JAKi; ≥ 18 years; palpable spleen ≥ 5 cm or SV ≥ 450 cm^3^	Median spleen volume of 1162 cm^3^	30/45 months	23/45	SVR35 (100%) at W24	Fatigue GI toxicity Anemia Leukopenia Thrombocytopenia	NCT04097821	[8]
PELA	BET	PO	II	One prior JAKi; ≥ 18 years; SV ≥ 450 cm^3^	Median age of 68 years	96/123 months	84/341	SVR35 (80%); MF-SAF-TSS50 (81%); BMF (40% ≥ 1 grade improvement) at any time	Thrombocytopenia Anemia	NCT02158858	[9]
Navitoclax	BCL-2 family	PO	II	One Prior IAKi or JAKi-naïve patients; ≥ 18 years; splenomegaly	JAKi-naïve pts; median age of 69 years; median SV of 1889 cm^3^	51/131 months	32/191	SVR35(59%) in high DIPSS score pts, BMF (35%) at W24	Not reported	NCT03222609	[10]
Parsaclisib	PI3K	PO	II	Pts with existing stable regimen of RUX; ≥ 18 years; palpable spleen ≥ 10 cm	Median age of 68 years; 47% of pts male; median SV of 2415 cm^3^ in QD/QW, 1878 cm^3^ in QD	63/63 months	74/74	SVR35 (7.1%); MPN-SAF-TSS50 (48.6%); BMF (33%) all daily dosing at W24	Pneumonia Fatigue Hypoxia Dyspnea Elevation of liver enzymes Hypocalcemia Thrombocytopenia	NCT02718300	[11]
SEL	XPO1	PO	I/II	JAKi-naïve pts; ≥ 18 years; SV ≥ 450 cm^3^	Median age of 64 years	17/44 months	19/237	SVR35 (84%) at any time; TSS50 (69%) at W12	Nausea Anemia Vomiting Thrombocytopenia	NCT04562389	[12]

## Conclusion

The next-generation JAKi have been evaluated in clinical trials, some even being FDA approved, to manage RUX intolerant or resistant MF. Other non-JAK/STAT therapeutic molecules, such as epigenetic modifiers, apoptotic machinery, and intracellular signaling pathway inhibitors, are also being investigated in clinical settings as both a single agent and in combination with RUX. The future of MF management is bright and promising.

## Data Availability

The material supporting the conclusion of this study has been included within the article.

## Abbreviations

ACVR1: Activin A receptor, type I
ASH: American Society of Hematology
BAT: Best Available Therapy
BET: Bromodomain and Extraterminal
BMF: Bone Marrow Fibrosis
HDM2: Human Double Minute-2
Hgb: Hemoglobin
JAK: Janus Kinase
JAKi: JAK inhibitor
LSD1: Lysine-specific demethylase-1
MF: Myelofibrosis
MMB: Momelotinib
MPN: Myeloproliferative Neoplasms
NA: Not available
NFκB: Nuclear Factor Kappa B
OS: Overall Survival
PELA: Pelabresib
PI3K: Phosphoinositide 3-kinase
PIM1: Proto-oncogene serine/threonine-protein kinase 1
PO: Per os (oral administration)
QD: Once Daily
QoL: Quality of Life
QW: Once Weekly
RP2D: Recommended Phase 2 Dose
R/R: Relapsed/refractory
RUX: Ruxolitinib
SIR: Siremadlin
STAT: Signal transducer and activator of transcription
SV: Spleen Volume
SVR: Spleen Volume Reduction
SAF: Symptom Assessment Form
SINE: Selective Inhibitor of Nuclear Export
SL: Spleen Length
SQ: Subcutaneous
TEAE: Treatment-Emergent Adverse Event
TI: Transfusion Independence
TI-R: Transfusion Independence Response
TSS: Total Symptom Score
VAF: Variant Allele Frequency
W12: Week 12
W24: Week 24
XPO1: Exportin-1

## References

[1] Hou JZ, Ye JC, Pu JJ (2021). Novel agents and regimens for hematological malignancies: recent updates from 2020 ASH annual meeting. J Hematol Oncol.

[2] Shantzer L, Berger K, Pu JJ (2017). Primary myelofibrosis and its targeted therapy. Ann Hematol.

[3] Gerds AT, Mesa R, Vannucchi AM, et al. Updated results from the momentum phase 3 study of momelotinib (MMB) versus danazol (DAN) in symptomatic and anemic myelofibrosis (MF) patients previously treated with a JAK inhibitor. In: 64th ASH annual meeting and exposition. New Orleans, Lousiana: American Society of Hematology; 2022:627.

[4] Oh S, Mesa R, Harrison C, et al. Pacritinib is a potent ACVR1 inhibitor with significant anemia benefit in patients with myelofibrosis. In: 64th ASH annual meeting and exposition. New Orleans, Louisiana: American Society of Hematology; 2022.

[5] Chaer F, McCloskey J, Rein L, et al. Preliminary Data from the Phase I/II Study of TP-3654, a Selective Oral PIM1 Kinase Inhibitor, in Patients with Myelofibrosis Previously Treated with or Ineligible for JAK Inhibitor Therapy. 64th ASH Annual Meeting and Exposition. New Orleans, Lousiana: American Society of Hematology; 2022:240.

[6] Pettit K, Gill H, Yacoub A, Bradley T. A phase 2 study of the LSD1 inhibitor bomedemstat (IMG-7289) for the treatment of advanced myelofibrosis (MF): updated results and genomic analyses. In: 64th ASH annual meeting and exposition. New Orleans, Louisiana: American Society of Hematology; 2022.

[7] Lu M, Xia L, Hoffman R. Pharmacological disruption of the interplay between HDM2-p53 and NFκB depletes myelofibrosis progenitor/stem cells and dampens the accompanying inflammatory Milieu. In: 64th ASH annual meeting and exposition. New Orleans, Lousiana: American Society of Hematology; 2022.

[8] Heidel F, Perkins A, Reiter A, et al. Siremadlin, a human double minute-2 (HDM2) inhibitor, added to ruxolitinib after suboptimal response to ruxolitinib alone in patients with myelofibrosis: results from part 1 of the phase 1/2 adore study. In: 64th ASH Annual Meeting and Exposition. New Orleans, Lousiana: American Society of Hematology; 2022.

[9] Mascarenhas J, Kremyanskaya M, Patriarca A, et al. Pelabresib (CPI-0610) combined with ruxolitinib for JAK inhibitor treatment-naïve patients with myelofibrosis: durability of response and safety beyond week 24. In: 64th ASH annual meeting and exposition. New Orleans, Lousiana: American Society of Hematology; 2022.

[10] Passamonti F, Foran J, Tandra A, et al. The combination of navitoclax and ruxolitinib in JAK inhibitor-Naïve patients with myelofibrosis mediates responses suggestive of disease modification. In: 64th ASH annual meeting and exposition. New Orleans, Lousiana: American Society of Hematology; 2022.

[11] Yacoub A, Borate U, Rampal R, et al. Efficacy and safety of add-on parsaclisib to ruxolitinib therapy in myelofibrosis patients with suboptimal response to ruxolitinib: final results from a phase 2 study. In: 64th ASH annual meeting and exposition. New Orleans, Lousiana: American Society of Hematology; 2022:236.

[12] Ali H, Kishtagari A, Maher K, et al. A Phase 1, open-label, dose-escalation study of selinexor plus ruxolitinib in patients with treatment-Naïve myelofibrosis. In: 64th ASH annual meeting and exposition. New Orleans, Lousiana: American Society of Hematology; 2022.

